# Laboratory Tests of Concrete Beams Reinforced with Recycled Steel Fibres and Steel Bars

**DOI:** 10.3390/ma14226752

**Published:** 2021-11-09

**Authors:** Małgorzata Pająk, Grzegorz Wandzik

**Affiliations:** Department of Structural Engineering, Silesian University of Technology, Akademicka 5, 44-100 Gliwice, Poland; grzegorz.wandzik@polsl.pl

**Keywords:** recycled steel fibres, flexure, concrete, DIC

## Abstract

This paper explores the possibility of the partial replacement of the longitudinal reinforcement in reinforced concrete (RC) beams with recycled steel fibres (RSF). Testing was focused on the contribution of two volume ratios of the RSF—0.5%, 1.0%. Basic compression and flexural tensile tests were performed to evaluate the effectiveness of the fibres following current standards. Additionally, the full-scale beams with and without conventional reinforcement were subjected to four-point bending tests. The results indicate that RSF improved the load-bearing capacity of the RC beams. Cooperation of RSF with the steel bars in carrying loads was proved. Findings from the Digital Image Correlation (DIC) revealed no impact on the cracking pattern of the RC beams.

## 1. Introduction

It is possible to reduce waste disposal amounts by their re-using in construction building materials. Currently, a huge problem all over the world is the disposal of end-of-life tires. In their reclamation process, two main components are obtained: crumb rubber and steel cord. The rubber component has found a wide range of applications in playgrounds, football pitches and urban pavements [1]. In the literature, attempts can be found to improve the mechanical properties of concrete with crumb rubber. It was found that the addition of rubber particles to concrete caused a pronounced reduction in compressive strength, mainly associated with the lower modulus of elasticity of the rubber in comparison to the concrete. It was shown that an addition of rubber, equal to around 20% of the aggregate content, reduced the compressive strength of concrete approximately by 50% [2,3]. The reduction of the basic characteristic of concrete is usually undesirable. Meanwhile, the second waste tire component consisting of a steel cord (recycled steel fibres) has been found to effectively reduce the brittleness of concrete, which is one of its weaknesses.

In general, randomly distributed short fibres improve the tensile strength and energy absorption capacity of concrete. The addition of fibres improve impact, fatigue, as well as fire and frost resistances of cement-based materials. Their effectiveness in the matrix strongly depends on the material (steel, polypropylene, glass, etc.), length and the shape (straight, hooked, crimped, etc.) [4,5]. The most effective are steel fibres, thus why various types of them are commercially available. The recycled steel fibres (RSF) from end-of-life tires differ from manufactured fibres with the stochastic length and diameter, and assorted shapes [6]. This is associated with the process of their reclamation and the type of the tires they are obtained from. Investigations on the impact of the RSF on mechanical properties of concrete have confirmed their usability to reinforce concrete [7,8,9,10,11,12,13].

Attempts to establish the contribution of different types of fibres in transferring loads in full-scale beams under flexure has been described in the literature [7,14,15,16,17,18].

The description of the research focused on the hooked-end fibres applied in the RC beams can be found in [15,17,18], for example.

RC beams with a span of 2.1 m made of concrete containing 40 kg/m^3^ and 60 kg/m^3^ of fibres were investigated in [15]. According to this study, in the beams with shear reinforcement and tensile reinforcement (*ρ* = 0.70%) the addition of fibres almost did not modify the mechanical response of the structural members. On the other hand, it is reported that in the beams where no stirrups were applied, the fibres lead to an increase of the shear strength.

Similar conclusions were found in [17], where the addition of hooked steel fibres (1%, 1.5%, 2%) did not lead to pronounced improvement of the load-bearing capacity in flexure. However, in the same experiments, the reduction in deflections at lower loads were reported. Concurrently, in [18] where the beams, with the span of 1.2 m and a reinforcement ratio *ρ* = 0.3–0.7%, were subjected to bending and the fibres led to a pronounced increase in load-bearing capacity. The cited publications lead to quite an obvious conclusion that weakly reinforced elements can be made more efficient with fibre reinforcement.

The comparable beam studies [7,14] were carried out for fibres derived from recycling. The basic difference in these types of fibres is related to their size and shape. Unlike manufactured fibres for dedicated use in concrete may have a shape depending on their acquisition and not fit as well as those manufactured. Several conclusions on RSF duplicate the conclusions formulated for those factory-produced. For example, the impact of the RSF, with volume ratios of 0.5% and 1.0%, on the behaviour of beams with the span equal to 3.42 m were investigated in [7]. Based on these studies, the positive influence of the RSF on the cracking phenomenon was indicating. The reduction of cracks width and their spacing was pointed out. Furthermore, it is confirmed that RSF are most effective in beams with lower reinforcement ratio. Additionally, the improvement of the load capacity, next to the reduction of the deflection and crack propagation was also mentioned in [14]. In that investigation the lathe fibres were applied with the volume ratio ranged from 0.5% to 2% to the RC beams with the span of 2.1 m.

Several attempts for substituting manufactured construction materials, used for reinforcing concrete beams (steel bars or fibres), with different types of natural or waste materials have been reported. The use of the bamboo fibres [19] or fibres from waste bottles [20] can be good examples. Their effectiveness still requires more investigation. The other example can also be the research providing that polypropylene discrete macro-fibres improved the ductility of beams reinforced with Glass Fibre Reinforced Polymer bars [16]. In this study, more cracks were noted distributed along the beams with fibres.

The investigation described in the present paper is focused on finding the contribution of recycled tire fibres on the bending behaviour of beams. To achieve this purpose, material and full-scale beam tests with concrete reinforced with conventional steel bars and two amounts of RSF were performed. Reference RC beams with no fibres added were also tested. Beams with small amounts of conventional reinforcement was chosen for full-scale tests, because they are shown to have a more pronounced influence of fibres addition [15,17].

## 2. Experimental Program

### 2.1. Materials

**Figure 1:**
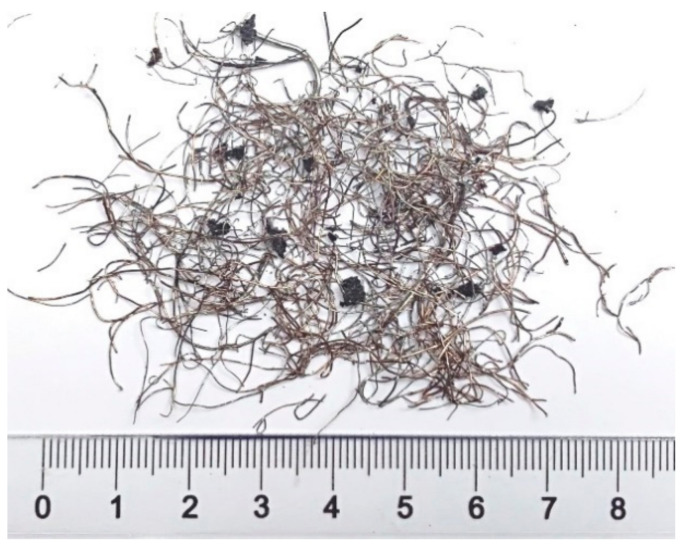
Fibres from the end-of-life tires used in the investigation.

The influence of RSF on the mechanical properties of the concrete matrix was studied. To achieve this, CEM III/A 42.5N—LH/HSR/NA cement produced by Górażdże Poland was used. The superplasticizer MasterSure 1200 produced by Master Builders Solutions Poland. (0.85% by mass of cement) was applied to maintain the concrete consistency. The exact composition of the concrete is presented in Table 1. The properties of fibres from the end-of-life tires produced by Gumeco Poland are summarized in Table 2. The fibres with a stochastic length and curved shape are shown in Figure 1. The same type of fibres were used by authors in previous investigations [11,12,13]. In the present investigation, the RSF were added to concrete in two amounts: 39.25 kg/m^3^ and 78.5 kg/m^3^ to create the recycled steel fibre reinforced concrete (abbreviation used in the manuscript—FRC). According to the above-mentioned works, these amounts guarantee mixes in which fibres can be used to partially replace conventional reinforcement. The accompanying tests of plain concrete with no fibres were also performed.

The slump flow tests of fresh mixes were performed according to PN-EN 12350-5 [21]. The addition of the RSF reduced the slump flow diameter (Table 3).

### 2.2. Specimens and Testing Methods

All specimens were cast in one concreting. The concrete was mixed in the concrete mixer and the fibres were added to it. The concrete was vibrated after being placed in the formwork. After one day the specimens were demoulded and cured in water at a temperature of 20 °C till the day of the tests.

#### 2.2.1. Compression and Flexural Tests

**Figure 2:**
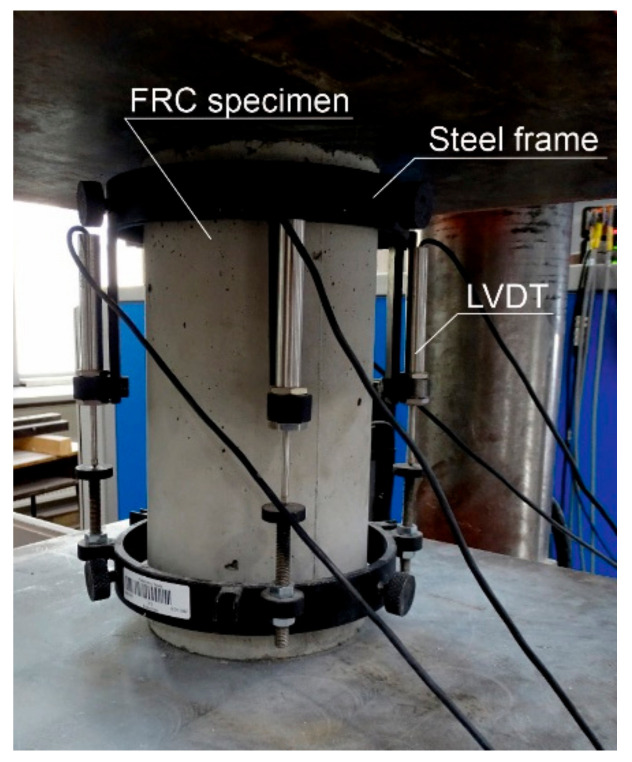
The compression test of the FRC specimen.

The compressive strength and modulus of elasticity were determined on cylindrical samples with a diameter of 150 mm and height of 300 mm. Six cylinders were prepared for each mix. The compression tests were carried out in a 3000 kN hydraulic testing machine according to EN 12390-3 [22]. The tests were performed applying a constant rate of displacement equal to 1 mm/min. The strains in the plain concrete specimens (mix C-0) were measured using 3 strain gauges. In the case of FRC, the strains were measured using the LVDTs mounted on the steel frame (Figure 2). For the FRC, the compression tests were performed with a constant rate of displacement to obtain the stress-strain curve after the achieving of the maximum load. This is a key, as the real influence of the fibres on the behaviour of the brittle matrix is observed in the post-peak regime of the stress-strain curve because the fibres bridge the cracks.

**Figure 3:**
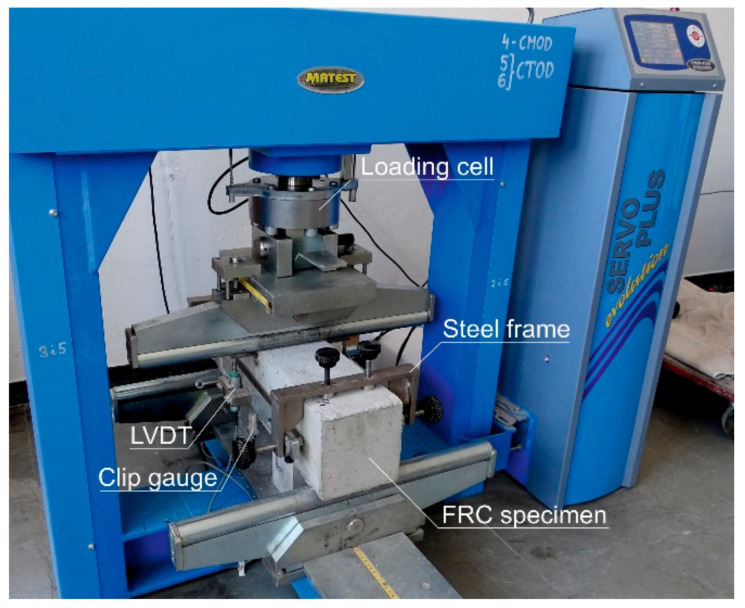
The flexural test of the FRC beam.

The flexural behaviour of FRC was analysed in a three-point bending test with notched beams with a dimension of 150 × 150 × 600 mm according to EN 14651:2007 [23]. The notch depth was equal to 25 mm. The flexural tests were performed on 4 specimens per mix. The deflection rate during the tests was equal to 2 mm/min. The tests were performed till the midspan-deflection reached 5 mm. The crack mouth opening displacement (CMOD) was measured during the tests. The view of the setup for flexural tests is presented in Figure 3.

#### 2.2.2. Structural Tests

**Figure 4:**
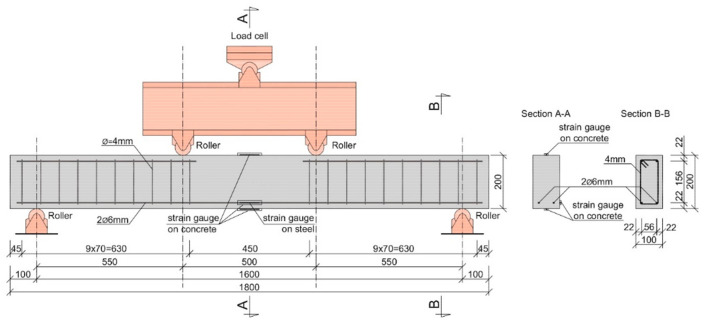
View of the RC/FRC-RC beam—four-points test layout.

The laboratory investigation also covered the full-scale beam tests. The height and the width of the beam was equal to 100 mm and 200 mm, respectively. The length of the beam was equal to 1800 mm (Figure 4). The testing of two beam types were performed: beams reinforced only with the recycled steel fibres (series FRC) and beams with both longitudinal and RSF reinforcement (series FRC-RC). The research program is presented in the Table 4**.**

**Figure 5:**
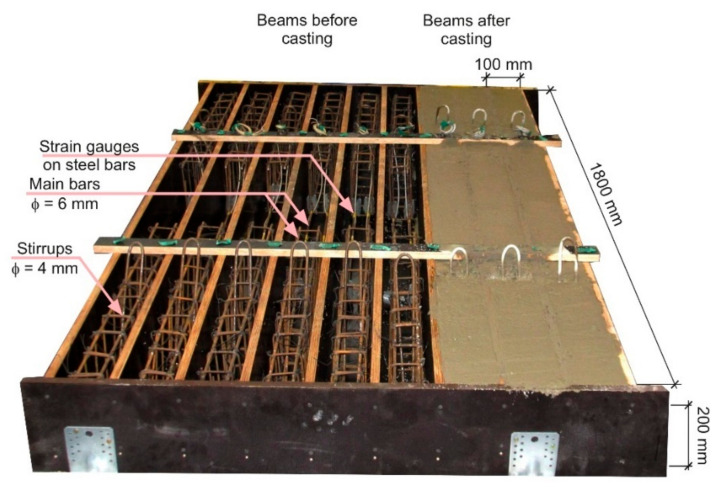
View of the RC beams before and after casting.

The RC beams were reinforced with two bottom longitudinal bars with a diameter of 6 mm and partially reinforced with upper longitudinal bars with a diameter of 6 mm at the support zones. The tensile reinforcement ratio was equal to 0.32%. The concrete cover was equal to 15 mm. The stirrups were only added in the shear zone of the beam, protecting it against shear failure. The shear reinforcement consists of stirrups with a diameter of 4 mm spaced every 70 mm (Figure 4). The strain gauges were mounted on the bottom steel bars at the midpoint of the beam. The view of the beams in the multi-station cast before and after casting is presented in Figure 5.

**Figure 6:**
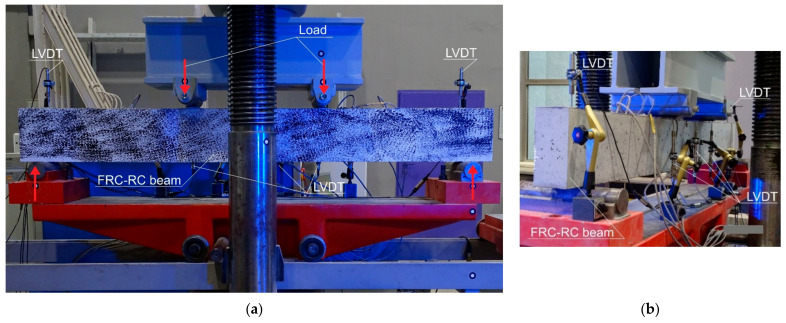
FRC-RC beams during the four-point test: (**a**) front view; (**b**) back view.

The beams with an overall length of 1.8 m were subjected to the four-point bending tests (Figure 6). The span of the beam was equal to *l* = 1.6 m. The load was applied with the constant rate of displacement equal to 1 mm/min till the failure of the specimen.

During the tests, the strains of the longitudinal bottom bars were measured. The strain of the beam surface was measured in the compression and tension zones. The strain gauges were glued to the beam—its arrangement is schematically presented in Figure 4. In the RC beams, the strain gauges were also placed on the side surface at the depth corresponding to the location of the longitudinal bars. The deflection of the beam in the middle and under the points where the load was applied were measured using LVDTs. The support positions were also controlled by the LVDTs.

The deformation of the almost whole front surface of the beam was monitored by the Digital Image Correlation (DIC) system—Aramis.

## 3. Results of Empirical Investigation

### 3.1. The Compression and Flexural Material Tests

**Figure 7:**
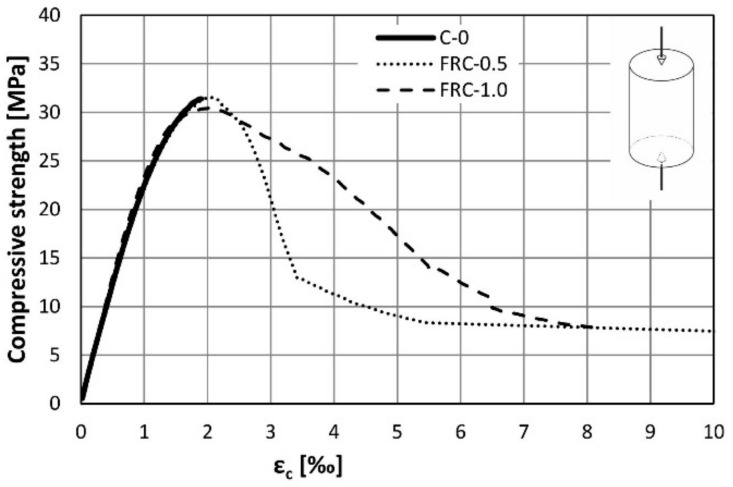
The stress-strain curves from the compression tests.

The results of the compression tests are presented in Table 5. The coefficient of variation (in percent) is shown in the parentheses. The addition of fibres had no noticeable effect on the compressive strength and the corresponding strain of the concrete. It confirms the expectations that the fibres slightly affect the compressive stress transfer mechanism. All average strain-stress curves are almost identical in the ascending part. Some influence of the fibres is visible on the post-peak part of the stress-strain curve—see Figure 7. The FRC exhibited the ability to carry the loads after achieving the peak point. The slope of the descending part of the curve was dependent on the fibre content, where an increase of the number of fibres was found to lower the softening rate.

**Figure 8:**
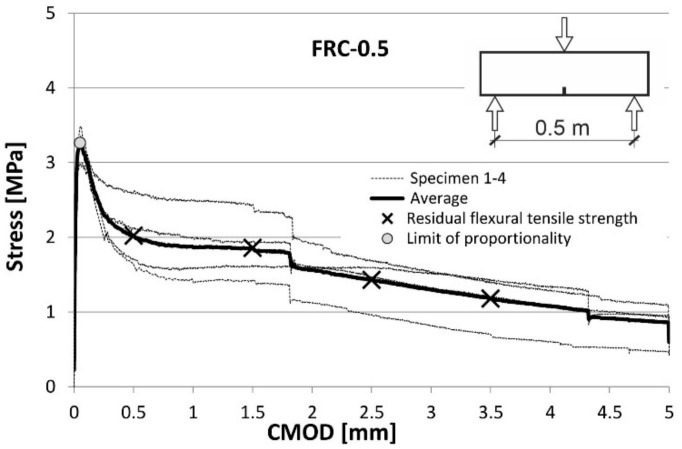
Stress-CMOD curves from flexural tests on FRC-0.5.

**Figure 9:**
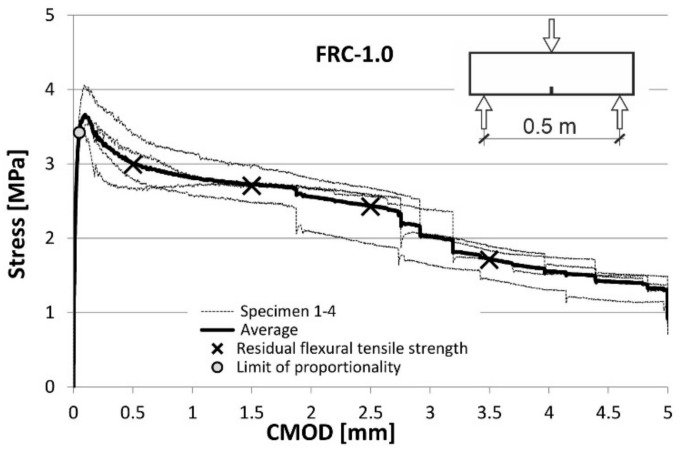
Stress-CMOD curves from flexural tests on FRC-1.0.

Fibre efficiency was determined in terms of their influence on the mechanical tensile properties of concrete. For this purpose, the FRC was tested in the three-point bending tests on notched beams [23,24]. The relationships between the tensile stress and CMOD established in these tests are shown for both types of FRC in the diagrams (Figure 8 and Figure 9) prepared for each test, as well as their average values.

The parameters obtained from the tests are the flexural tensile strength at the limit of proportionality (*f_L_*) and the residual flexural tensile strengths (*f_R,i_*) at the four values of crack mouth opening displacement (CMOD): 0.5 mm, 1.5 mm, 2.5 mm and 3.5 mm. All these values are presented in Figure 8 and Figure 9. A description of the method applied for stress calculation can be found in another author’s publication [11]. All basic tensile properties are summarized in Table 6.

To determine the possibility of the partial replacement of conventional reinforcement with fibres, the Model Code 2010 [25] proposes an analysis of *f_L_* together with *f_R._*_1_ and *f_R_*_.3_ according to Equation (1):

*f_R._*_1_/*f_L_* > 0.4; *f_R_*_.3_ / *f_R_*_.1_ > 0.5 (1)

Furthermore, according to Model Code 2010 [25], the constitutive law for FRC under tension can be derived using the parameters mentioned above. On the other hand, the standard EN 14889-1:2006 [26] specifies the minimum residual strength values that have to be achieved to consider the fibres as effective (2):

*f_R_*_.1_ > 1.5 MPa; *f_R_*_.4_ > 1.0 MPa (2)

A simple comparison of the diagrams in Figure 8 and Figure 9 shows that an increase in the fibre content (in the tested range) clearly improves the strength properties of concrete in tension. At the same time, the requirements expressed in formulas (1) and (2) are met (see Table 6). This indicates that according to Model Code 2010 [25] the tested fibres are potentially able to partially replace the conventional reinforcement.

### 3.2. The Full-Scale Beam Tests

#### 3.2.1. The Beams Reinforced Only with the RSF

**Figure 10:**
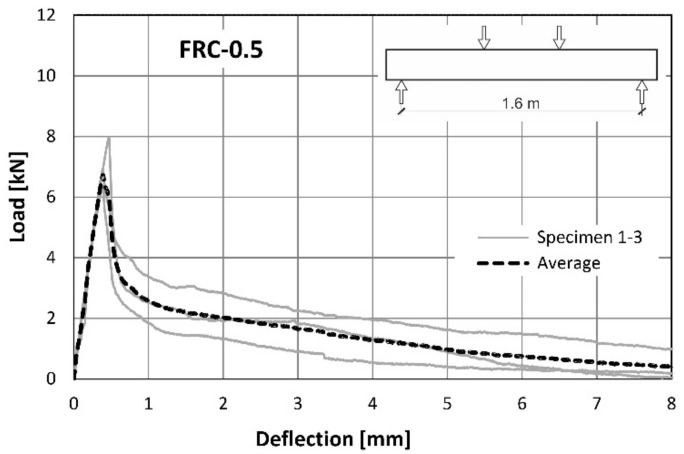
Load–deflection curves from flexural tests on FRC-0.5.

**Figure 11:**
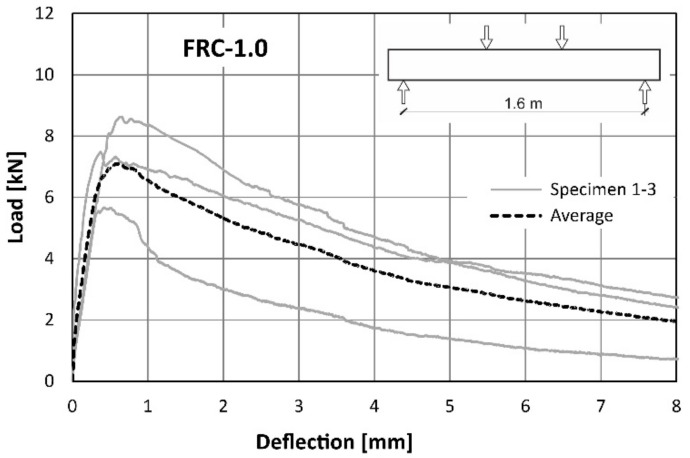
Load–deflection curves from flexural tests on FRC-1.0.

The load-deflection curves obtained in the flexural tests (described in the chapter 2.2.2) performed on the full-scale beams reinforced only with the recycled steel fibres are presented in Figure 10 and Figure 11 or FRC-0.5 and FRC-1.0, respectively. The tests were performed till the mid-span deflection reached the value of *l*/200 = 8 mm.

**Figure 12:**
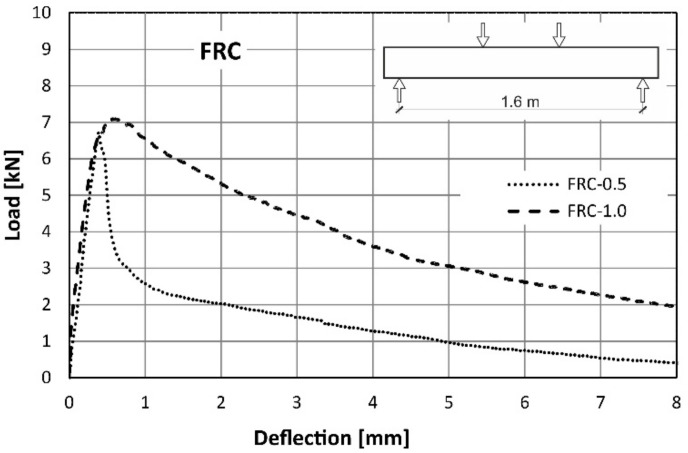
Comparison of the load-deflection curves from flexural tests on FRC series.

Direct comparison of the average curves presented in Figure 10 and Figure 11 is also presented in the Figure 12. It can be concluded that the load-bearing capacity of both types of beams was comparable. The slightly higher value for the maximum load was noted for the beams with the higher amount of the fibres (Table 7). The mid-span deflection corresponding to the maximum load was two times bigger for FRC-1.0 than for FRC-0.5 (Table 7). However, in the case of FRC-1.0, a higher scatter of results was observed (Figure 11). The influence of both amounts of fibres was noted in the post-peak regime of the load-deflection curve. Generally, in the case of samples reinforced with RSF (FRC-0.5), a significant softening after reaching the peak load was noted. The higher number of fibres (FRC-1.0) caused some hardening before the maximum load capacity was achieved and mild softening in comparison to FRC-0.5. FRC beams with a higher fibre content were found to withstand the load carrying capacity much longer. This is clearly related to FRC’s ability to retain tensile stress after cracking. For example, when the deflection reached the value of 2 mm, the load applied to the FRC-0.5 beam was 2.0 kN, while for FRC-1.0 it was more than twice as much and amounted to 5.3 kN. This confirms that an increase in the number of fibres, leads to an increase of efficiency in the post-peak phase. The detailed results of the structural tests are summarized in Table 7.

**Figure 13:**
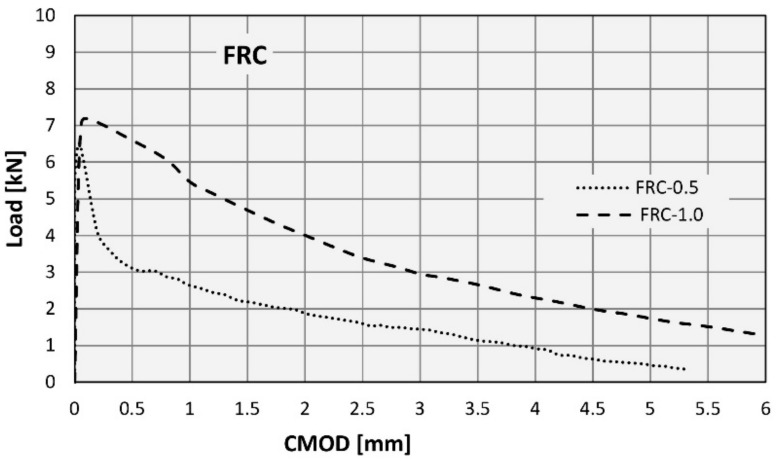
The load-CMOD relationship determined with the use of DIC.

**Figure 14:**
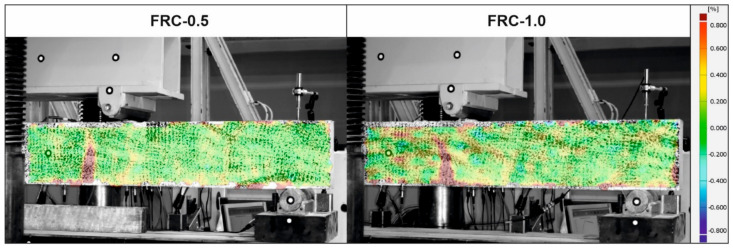
Distribution of the principal strains on the front surface of the beams based on DIC.

The course of the tests was monitored with the use of DIC which determined the development of cracks in beams. The CMOD presented in Figure 13 represents the evolution of the main cracks in the beams. They are the average of CMOD values obtained for three FRC-1.0 beams and one for the FRC-0.5 beam. In two other FRC-0.5 beams the main crack appeared in the zone not monitored by the DIC. Please note that because of technical limitations only the half of the beam could be observed. The results confirmed the better capacity of the FRC-1.0 beams to withstand loads than the FRC-0.5. The comparison of the views of the front surface of the selected beams monitored by the DIC are presented in Figure 14. The distribution of the principal strains at the moment when the CMOD is equal to around 0.2 mm is presented in Figure 14. In both beams only one crack was formed till the failure of the beam. However, it can be seen that for 0.5% of the fibres, the principal tensile strains are localised closer to the crack, while when more fibres are used (FRC-1.0) principal strains are more distributed in the whole zone of the constant bending moment, not only near the crack.

#### 3.2.2. Beams Reinforced with Fibres and Longitudinal Reinforcement

**Figure 15:**
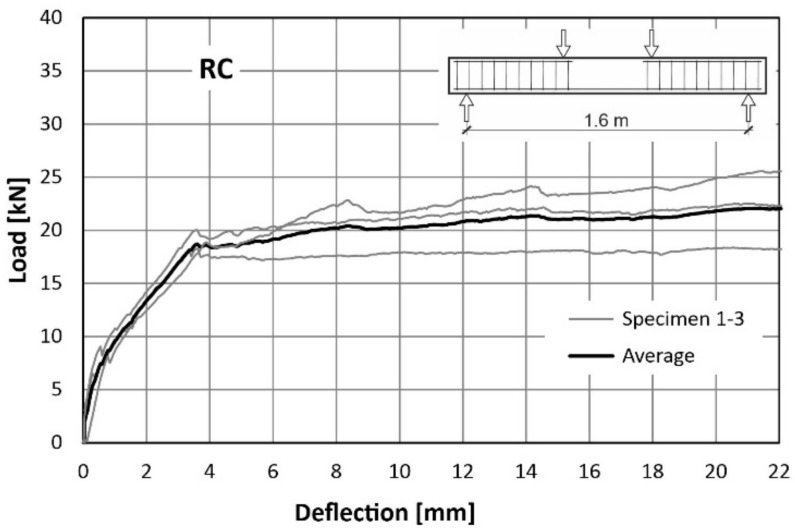
Load-deflection curves from flexural tests on RC beams.

**Figure 16:**
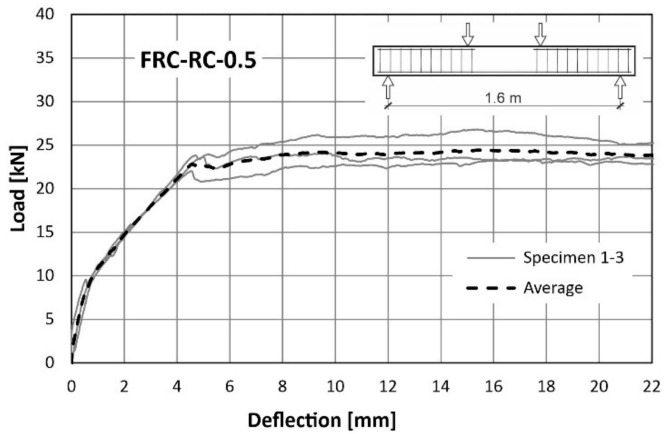
Load-deflection curves from flexural tests on FRC-RC-0.5.

**Figure 17:**
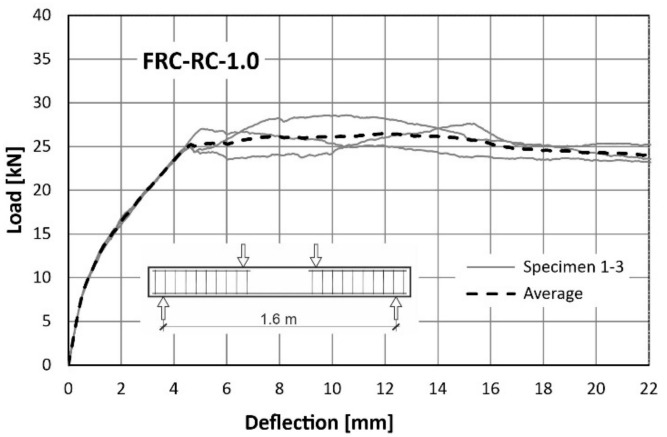
Load-deflection curves from flexural tests on FRC-RC-1.0.

**Figure 18:**
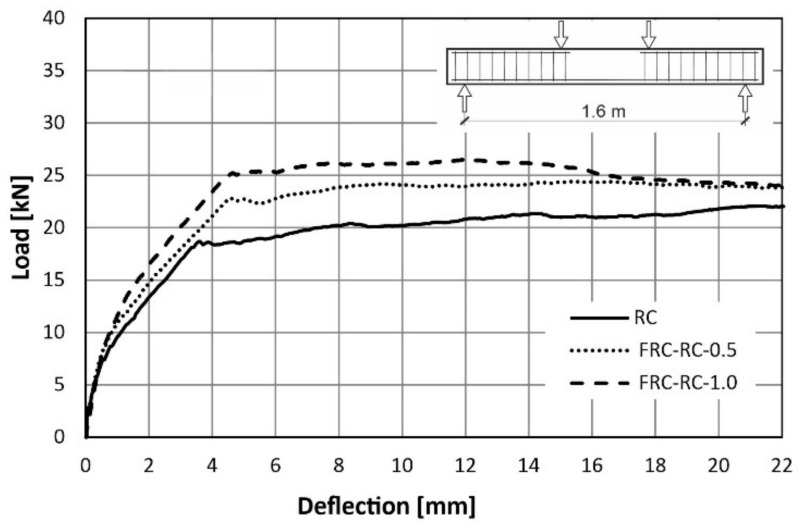
Comparison of the load-deflection curves from flexural tests on FRC-RC series.

The behaviour of the tested beams was evaluated using load-deflection curves. The first plot (Figure 15) was made for a reference RC beam (reinforced with traditional reinforcement only), while next two (Figure 16 and Figure 17) for FRC-RC beams reinforced with both—fibres and traditional reinforcement. The aforementioned graphs show the dependence for all beams in the series and the average dependence as well. Figure 18 is a summary of all average load-displacement relationships for beams with different fibre contents. All relationships were presented until a deflection of 22 mm was achieved.

Generally, the course of the load-deflection curves for all three beams in each series have a similar shape. In each case, three sections can be distinguished. The boundaries between them are the result of the first cracks appearing and the reinforcing steel yielding.

Along the first segment (until the deflection of about 0.4 mm is achieved), the analysed dependence is the same for all 3 series. In this range, the beams work elastically and there is no pronounced influence of the fibres. It begins to be visible after exceeding the load of about 8 kN (corresponding to the appearance of the first cracks). The load-deflection curve was flattened above this load level, which led to the more pronounced increase of deflections in all cases. Nevertheless, this was seen to be faster with the lower fibre content. For a force of 16 kN, the average deflections were: 2.77 mm (RC), 2.38 mm (FRC-RC-0.5) and 1.89 mm (FRC-RC-1.0).

The merit of the use of the randomly distributed fibre reinforcement is resulted in an increase in the load-bearing capacity. For the FRC-RC-1.0 series the maximum load was 20% higher than for the RC series and reached 27.77 kN (compared to 22.93 kN for RC beams).

However, analysing the whole course of the load-deflection curve it can be noted that the benefit of the fibres decreased with increasing deflection. This led to the increasingly greater plastic deformation of the reinforcing steel bars and the progressive opening of cracks which decreased in the tensile transmission by fibres. This is manifested in the slow re-approach of the three curves in Figure 18 for large deflections. Thus, it can be concluded that the benefit of the fibres is reduced at very small and very large beam deflections (see Figure 12 and Figure 13). The maximum loads (averaged for all beams in series) and the corresponding deflections can be found in Table 7.

**Figure 19:**
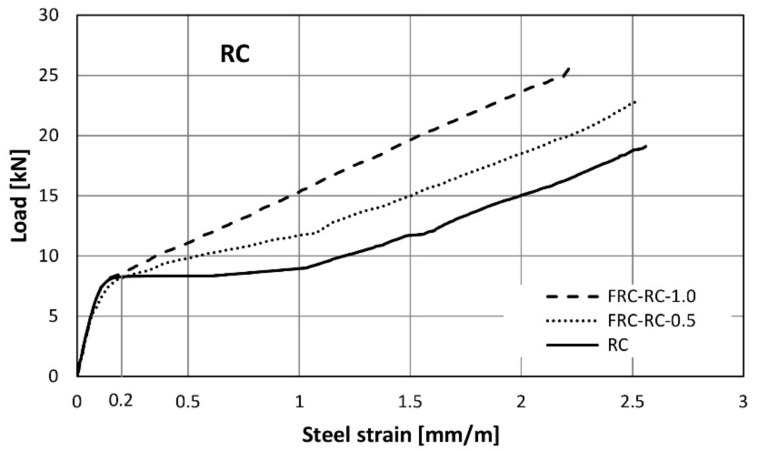
Strain in bottom steel reinforcement in RC/FRC-RC beams.

The strain gauges placed on the bottom bars measured steel elongation for the duration of the test. Figure 19 shows the variability of the average steel strain (average of two bars and three beams in the series) with increased loading. The course of the curves shows that the fibres begin to actively work when the strain in the steel reaches 0.2‰, which corresponds to the tensile strength of concrete.

After exceeding a load of 8 kN (strain about 0.2‰), the rate of strain in steel became much faster. This is a typical effect for reinforced concrete elements resulting from the loss of the ability to transmit tensile stresses by concrete in the cross-section coming through the crack. Above this load level, a clear differentiation in the functioning of the beams depending on the fibre content can be observed. When analysing any force level after cracking, it can be seen that in the elements made of plain concrete, the steel strains are significantly greater. The need to ensure an equilibrium of forces in the cross-section together with the lower steel strains and stresses clearly indicate the participation of fibres in the transmission of tensile forces in the cross-section. For example, analysing the strains (Figure 19) it can be concluded that for the load *F* = 15 kN the tensile force transmitted by the reinforcement in the FRC-RC-1.0 beam is only half that for the element without fibres (RC).

It is also worth paying attention to the course of the graph immediately after cracking (in the force range of 8–10 kN). In an element without fibres, the increase in steel strain is rapid and occurs at a constant load value. This shows the high speed of the cracking process in this type of element and the rate with which unreinforced concrete loses its tensile capacity. In the tests, this strain increment took place in a very short time. The behaviour of the elements with fibres is slightly different. After the crack is formed, the steel elongates gradually and is accompanied by a slight increase in force (greater for elements with higher fibre content). Based on this, it is possible to reduce the abruptness of the change in the strain state in the cross-section.

The diagram in Figure 19 shows the deformation of steel bars only in the elastic phase. After rapid change in steel strain, in range from 0.2‰ to 1‰ in RC elements, a further increase in load is associated with a gradual elongation. From a strain of about 1‰, all three lines (representing different fibres content) are almost parallel. This indicates the constant cooperation of the RSF with the conventional bars in this domain.

**Figure 20:**
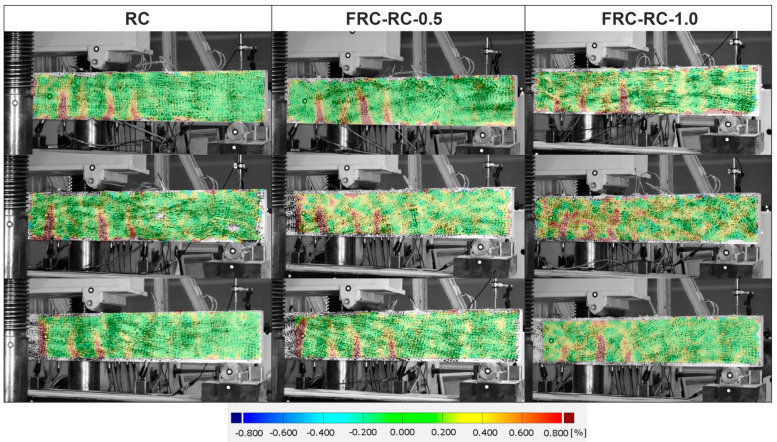
Distribution of the principal strain on the front surface of the RC/FRC–RC beams based on DIC.

**Figure 21:**
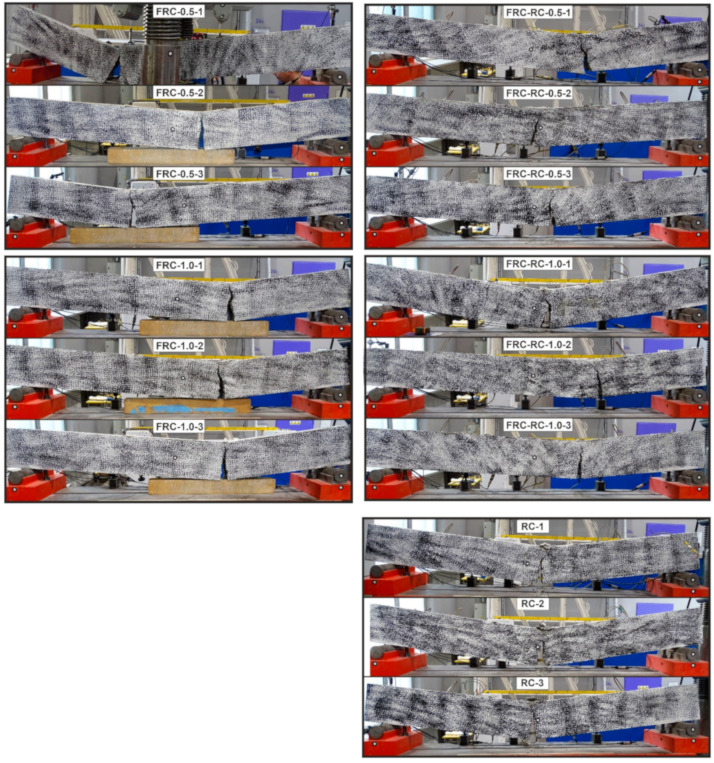
The view of the beams after the test.

During the tests, more than half of the side surface of the beams was continuously monitored by the DIC system. The distribution of the principal strains in all 9 reinforced beams is presented in Figure 20. The images shown in Figure 20 correspond to the load when the steel strain reached the value of 0.2‰, when crack formation in each beam become visible. The comparison of these images shows that the presence of the fibres has no significant effect on the number of major cracks and the distance between them. Nevertheless, the increase of fibre volume ratio resulted in the distribution of the strains on the side surface of the beam to change. The tensile zones also appeared in the middle part of the beams reinforced with the fibres. It demonstrates that, due to the fibres, the whole beam takes part in carrying the loads. In the next steps the opening of one crack was observed in all the beams. The view of all the beams after the test is presented in Figure 21.

## 4. Conclusions

The volume ratios of 0.5% and 1.0% of recycled steel fibres improved the tensile parameters of the concrete matrix what was proved based on the flexural tensile tests on notched beams. The residual flexural tensile strength values obtained indicated that the RSF can be used to partially replace conventional reinforcement. The increase of flexural strengths was proportional to the increase of the number of fibres. No apparent influence of the fibres on the compressive strength of concrete was noted.

This study carried out using full-scale beams reinforced only with the recycled steel fibres or their combination with longitudinal reinforcement indicate that:

- The addition of the fibres resulted in an increase of the load-bearing capacity of the RC beams. The maximum load went from 22.93 kN on average for RC beams to 27.77 kN on average for the FRC-RC-1.0 beams This demonstrates the efficiency of the fibres even at the ultimate limit state. For the same load, the strains in steel bars were lower in the beams where RSF were applied;
- In the beams with a 1.6 m span the application of RSF had no apparent influence on the distance between the cracks and their number;
- The beams reinforced only with the RSF were able to carry the loads after cracking. The maximum load recorded was equal to around 7 kN. Only one crack was noted in the brittle failure of all FRC beams.

## Figures and Tables

**Table 1:** Composition of concrete mix.

Mix	Cement [kg/m^3^]	Sand [kg/m^3^]	Coarse Aggregate [kg/m^3^]	Silica Fume [kg/m^3^]	Superplasticizer	Water [L/m^3^]	Fibres	
							[kg/m^3^]	Volume Ratio *V_f_*
C-0	300	840	892	80	2.55	165	0	0
FRC-0.5							39.25	0.5%
FRC-1.0							78.5	1.0%

**Table 2:** RSF properties.

Length [mm]	Diameter [mm]	Tensile Strength [MPa]	Aspect Ratio (Length/Diameter)	Longitudinal Shape
~2–50	0.15 ± 5%	≥2850	13–200	irregular (curved, twisted)

**Table 3:** Slump flow.

Mix	Fibres Volume Ratio *V_f_*	Slump Flow [mm]
C-0	0	495
FRC-0.5	0.5%	440
FRC-1.0	1.0%	375

**Table 4:** Research program.

Mix	Fibres Reinforcement—Fibres Volume Ratio *V_f_*	View of the Beam	Longitudinal Reinforcement—Bars	Shear Reinforcement—Stirrups	Number of Beams
FRC-0.5	0.5%	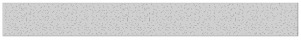	-	-	3
FRC-1.0	1.0%		-	-	3
RC-0	0	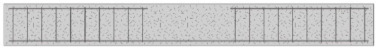	2 ø 6 mm	ø 4 mm every 70 mm	3
FRC-RC-0.5	0.5%		2 ø 6 mm	ø 4 mm every 70 mm	3
FRC-RC-1.0	1.0%		2 ø 6 mm	ø 4 mm every 70 mm	3

**Table 5:** Results from the compression tests.

Mix	Fibres Reinforcement—Fibres Volume Ratio *V_f_* [%]	Compressive Strength [MPa]	Strain Corresponding to Compressive Strength [10^−3^]
C-0	0	31.36 (4)	1.9 (7)
FRC-0.5	0.5	31.76 (2)	2.1 (5)
FRC-1.0	1.0	30.82 (5)	1.9 (19)

**Table 6:** Three-point flexural tensile tests—mean values.

Mix	Fibres Reinforcement—Fibres Volume Ratio *V_f_* [%]	Limit of Proportionality [MPa]	Residual Flexural Tensile Strength [MPa]				*f_R_*_.3_/*f_R_*_.1_	*f_R_*_.1_/*f_L_*
		*f*_L_	*f*_R.1_	*f*_R.2_	*f*_R.3_	*f*_R.4_		
C-0	0	2.95	-	-	-	-	-	-
FRC-0.5	0.5	3.26	2.02 > 1.5	1.86	1.43	1.18 > 1.0	0.71 > 0.5	0.62 > 0.4
FRC-1.0	1.0	3.42	2.99 > 1.5	2.7	2.43	1.71 > 1.0	0.81 > 0.5	0.87 > 0.4

**Table 7:** Results from the flexural tests on beams.

Mix	Maximum Total Load [kN]	Bending Moment [kNm]	Deflection Corresponding to the ε_c_ = 0.2‰
FRC-0.5	7.09 (1)	1.95	0.3
FRC-1.0	7.26 (1)	2.00	0.72
RC	22.93 (5)	6.31	3.61
FRC-RC-0.5	24.84 (2)	6.83	4.57
FRC-RC-1.0	27.77 (1)	7.64	4.64

## Data Availability

The data presented in this study are available on request from the corresponding author.
